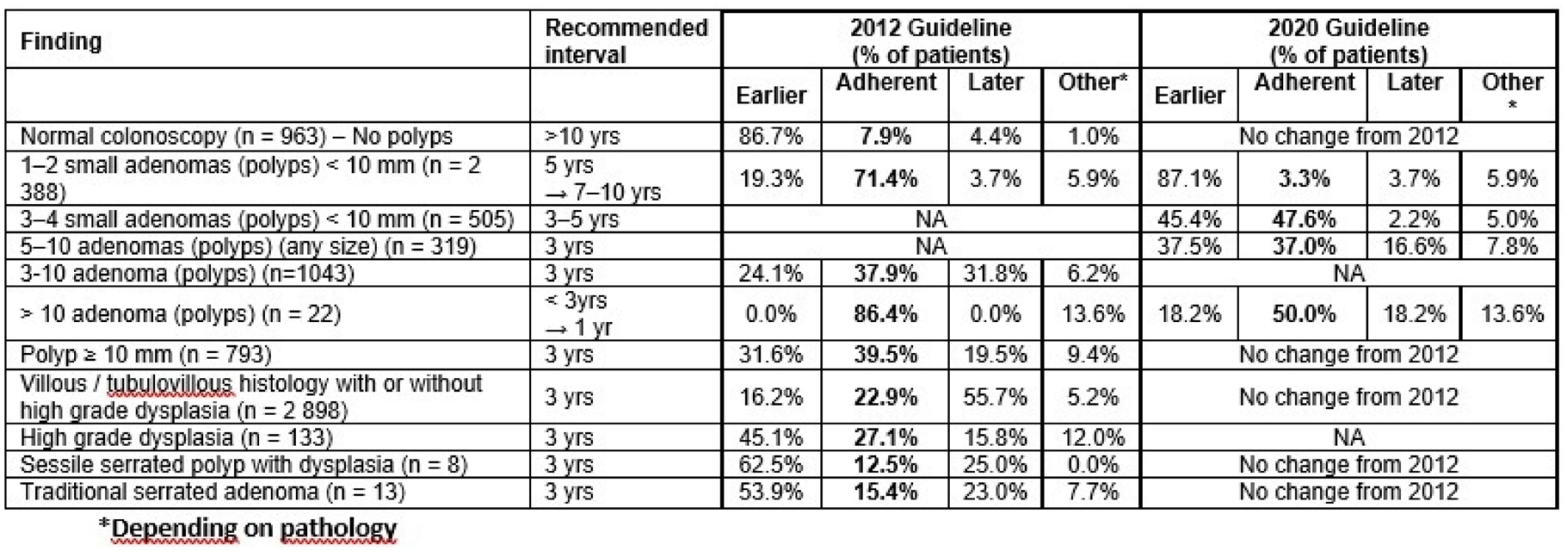# Poster Session I – Poster of Distinction - A96 ADHERENCE TO PAST AND CONTEMPORARY POST-POLYPECTOMY SURVEILLANCE GUIDELINES

**DOI:** 10.1093/jcag/gwaf042.096

**Published:** 2026-02-13

**Authors:** C Hansen-Barkun, S Aprikian, N Milky, K Shanahan, J Beauchesne-Blanchet, M Martel, C Menard, D Von Renteln, A N Barkun

**Affiliations:** McGill University, Montreal, QC, Canada; McGill University, Montreal, QC, Canada; Research Institute of the McGill University Health Centre, Montreal, QC, Canada; Research Institute of the McGill University Health Centre, Montreal, QC, Canada; Research Institute of the McGill University Health Centre, Montreal, QC, Canada; Research Institute of the McGill University Health Centre, Montreal, QC, Canada; Universite de Sherbrooke, Sherbrooke, QC, Canada; Universite de Montreal, Montreal, QC, Canada; McGill University, Montreal, QC, Canada

## Abstract

**Background:**

United States MultiSociety TaskForce (USMSTF) guidelines addressing post-polypectomy colonoscopy intervals were issued in 2012 and modified in 2020.

**Aims:**

Assess post-polypectomy surveillance intervals adherence to USMSTF 2012 and 2020 recommendations.

**Methods:**

We included patients with a past personal or index colonoscopy history of colon polyps from two tertiary hospitals. Procedural reports were reviewed and patients stratified according to index colonoscopy findings into: normal colonoscopy, 1–2 small (<10 mm) adenomas, 3–4 small adenomas, 5–10 polyps, >10 polyps, ≥1 adenoma ≥10 mm, at least one adenoma with high grade dysplasia, or a serrated polyp with dysplasia, or a traditional serrated adenoma. Recommended follow-up intervals were classified as earlier, later or adherent to 2020 guidelines, and compared to 2012 recommendations.

**Results:**

Overall, 5,204 colonoscopies were included over 47 months. When the first follow-up index colonoscopy was normal (n = 963), adherence to follow-up was only 7.9% as per both guidelines, i.e.: 86.7% scheduled sooner than recommended (<10 years). For patients with 1–2 adenomas (<10 mm) (n = 2,388), adherence was 71.4% according to 2012 guidelines (5 years) but fell to 3.3% with 2020 recommendations (7-10 years); most patients were still assigned a 5-year follow-up (87.1%). For 3–4 small adenomas (<10 mm) (n = 505), adherence was 47.6% for 2020 guidelines (3-5 years). When >10 polyps were removed (n = 22), there was better compliance, with adherence rates of 86.4% according to 2012 guidelines (< 3 years) and 50.0% for the 2020 recommendations (1 year). For lesions requiring a 3-year surveillance interval common to both guideline era, adherence was 39.5% for polyps ≥10 mm, 22.9% for villous/tubulovillous histology with/without high grade dysplasia, 27.1% for adenoma with high grade dysplasia, 12.5% for sessile serrated polyps with dysplasia, and 15.4% for traditional serrated adenomas. All non-adherent recommendations suggested a colonoscopy sooner than the recommended intervals, except for those with villous/tubulovillous histology with or without dysplasia, among whom 55.7% were later than the recommended post-polypectomy colonoscopy intervals.

**Conclusions:**

Continued practice according to earlier-surveillance interval persist, particularly for low-risk patients with a normal colonoscopy or 1–2 small adenomas, despite the revises 7–10 year extended interval recommendations in 2020. Adherence for high-risk findings was moderate and unchanged, while delayed follow-up was most frequent among patients with villous or tubulovillous adenomas. There is a need for improved implementation of societal recommendations to ensure optimal evidence-based surveillance practices in the face of what are often limited colonoscopy resources.

**Funding Agencies:**

The study as been funded by the CPAC